# *In Vitro* and *In Vivo* Interventions Reveal the Health Benefits of Levan-Type Exopolysaccharide Produced by a Fish Gut Isolate *Lactobacillus reuteri* FW2

**DOI:** 10.3390/life15010089

**Published:** 2025-01-13

**Authors:** Waqar Ahmad, Anam Nasir, Satya Prakash, Azam Hayat, Mujaddad ur Rehman, Shazia Khaliq, Kalsoom Akhtar, Munir Ahmad Anwar, Nayla Munawar

**Affiliations:** 1 Industrial Biotechnology Division, National Institute for Biotechnology and Genetic Engineering College, Pakistan Institute of Engineering and Applied Sciences (NIBGE-C, PIEAS), Faisalabad 38000, Pakistan; waqarahmadhu424@gmail.com (W.A.); anam_nasir1991@yahoo.com (A.N.); skhaliq1976@gmail.com (S.K.); kalsoom1967@gmail.com (K.A.); 2 Biomedical Technology and Cell Therapy Research Laboratory, Department of Biomedical Engineering, Faculty of Medicine, McGill University, 3775 University Street, Montreal, QC H3A 2B4, Canada; satya.prakash@mcgill.ca; 3 Department of Microbiology, Abbottabad University of Science and Technology, Havelian, Abbottabad 22500, Pakistan; azamhayyat@yahoo.com (A.H.); mujaddad@aust.edu.pk (M.u.R.); 4 Department of Chemistry, College of Sciences, United Arab Emirates University, Al-Ain 15551, United Arab Emirates

**Keywords:** levan, prebiotics, intestinal health, antioxidant, *Lactobacillus*

## Abstract

Microorganisms synthesize diverse types of exopolysaccharides (EPSs). EPSs with varying structural and physical properties can demonstrate unique health benefits, which allow for their tailored applications as functional foods such as prebiotics. Levan, a fructose-based EPS, is gaining considerable attention as an effective prebiotic to support the growth of beneficial gut bacteria. Consequently, this enhances digestive health, boosts the immune system, and reduces the risk of chronic diseases. Unfortunately, limited studies are available on levan-type EPSs to demonstrate their role as prebiotics. Therefore, in this study, we conducted in vitro and in vivo experiments, concerning intestinal cell integrity and metabolic syndrome, to assess the therapeutic potential of levan derived from *Lactobacillus reuteri* FW2. The in vitro experimental results revealed that levan improved the survival of impaired HT-29 epithelial cells of the intestine and also exerted antioxidant effects. In the in vivo experiments, mice fed with levan-supplemented feed exhibited low body weight gain, blood glucose, and serum cholesterol levels compared to the control group. These findings highlight the biotherapeutic potential of *L. reuteri* FW2-derived levan for improving metabolic syndrome and its associated aspects. It also signifies the need for a further detailed investigation based on clinical trials to include levan in dietary supplements for improved health and well-being.

## 1. Introduction

A remarkable number of commensal bacteria residing in the human gastrointestinal tract exist in a state of homeostasis with their host [1,2]. The symbiotic relationship established with the host through the presence of the microbiota exerts a profound impact on the host’s health. This influence is demonstrated through various mechanisms such as nutrient provision, metabolite conversion, regulation of epithelial cell proliferation, prevention of pathogen growth, lowering cholesterol, and enhancing innate and/or adaptive immunity [3]. Consequently, there is considerable interest in exploring non-digestible food components that can induce specific changes as well as promote the growth of beneficial microbiota [4]. In this context, prebiotics are emerging as non-digestible food additives that stimulate the growth and activity of intestinal bacteria, primarily bifidobacteria, lactobacilli, and Bacteroides, thereby leading to the improvement in the overall health of the host [5].

Research interests focused on discovering natural polysaccharides with desirable properties for application as prebiotics represent a crucial area of investigation worldwide [6,7,8,9]. Among these natural polysaccharides, levan stands out as a promising candidate for diverse applications across various industries, including medicine, food, agriculture, aquaculture, consumer personal care products, and particularly in the realm of prebiotics [10,11,12]. Levan is a non-toxic, biocompatible, water-soluble, and film-forming polysaccharide, characterized by β-(2-6) linkages and a non-structural homopolymeric composition [13]. Levan is known to be produced by some plants and several Gram-positive and Gram-negative bacterial species [14]. Microbial levan’s value and versatility as a biopolymer stem from its biocompatibility and simple production process [15]. Hamdy et al. [16] proposed a potential link between levansucrase-producing bacteria and their prebiotic and probiotic attributes.

The potential of levan to improve host health can be attributed to its prebiotic behavior. For instance, levan has demonstrated capabilities to resist degradation in the upper gastrointestinal tract and selective utilization by gut microbiota in the large intestine [17,18]. Similarly to traditional prebiotics, the fermentation of levan produces short-chain fatty acids (SCFAs) such as acetate, propionate, and butyrate. The production of SCFAs is a marker of a healthy gut ecosystem, which ensures the proper functioning of the intestines, reduces the risk of gut dysbiosis, and accelerates the process of the healing and regeneration of the intestinal epithelium [19]. Produced SCFAs are also transported to other organs of the body where they demonstrate several benefits, such as antioxidant, immunomodulatory, anti-obesity, anti-cancerous, and cholesterol, lipid, and blood glucose modulating properties [20].

Despite the considerable potential that levan has demonstrated as a versatile polymer, there are only a limited number of studies in the literature that have explored its possible implications for human gastrointestinal health. Notably, some in vitro studies have demonstrated the potential modulatory effect of levan on colonic microbiota, but very few researchers have studied its efficacy in vivo [21,22,23,24]. Driven by the limited available research on the health benefits of levan, this study aims to provide the first systematic investigation of the in vivo effects of levan on metabolic syndrome in mice models, and in vitro effects on human intestinal epithelial cells.

In the current study, levan from a previously isolated *Lactobacillus reuteri* FW2 [25] was used to explore its health-beneficial effects through in vitro and in vivo experiments. For in vitro studies, human HT-29 intestinal epithelial cells were used to study the potential of levan on cell viability, cytotoxicity, and reactive oxygen species (ROS). For in vivo studies, experimental mice were used to investigate the effects on body weight gain, blood glucose, and serum cholesterol levels. The overall results exhibited the biotherapeutic potential of *L. reuteri* FW2 levan for the improvement in dysmetabolic syndrome.

## 2. Materials and Methods

### 2.1. Bacterial Strain and Biosynthesis of EPS

#### 2.1.1. Bacterial Strain

*L. reuteri* FW2, an isolate that synthesizes levan-type exopolysaccharides (EPSs), was previously isolated from the gut of local fresh-water fish (*Tor putitora*) and identified, and its EPS was characterized by our lab [25]. In the present study, the characterized levan is used to further investigate its potential health benefits by in vitro and in vivo approaches.

#### 2.1.2. Biosynthesis of EPS

Levan from *L. reuteri* (FW2) was produced according to the previously described method [26]. Briefly, the isolate *L. reuteri* FW2 was cultured in liquid MRS (supplemented with 20% sucrose) medium for 72 h at 37 °C. After obtaining growth, the culture was centrifuged at 6000× *g* for 15 min. Subsequently, the supernatant was treated with 4% *v*/*v* trichloroacetic acid (TCA) solution and kept on a shaker for 45 min at 100 rpm to precipitate all the proteins. The incubation was followed by centrifugation at 6000× *g* for 15 min and the collection of the supernatant. Two volumes of ice-cold absolute ethanol were added to the supernatant for the precipitation of the EPS. The precipitated EPS was collected by centrifugation at 6000× *g* and washed twice with ice-cold ethanol to remove mono- and di-saccharides. All the centrifugations were performed at 4 °C. The purified levan was dissolved in dH_2_O and lyophilized for use in further experiments.

### 2.2. In Vitro Experiments

#### 2.2.1. Cell Culturing

HT-29 human intestinal cells were grown in McCoy’s 5A medium, enriched with 10% fetal bovine serum (FBS; Thermo Fisher Scientific, Waltham, MA, USA). The cells were cultured within an environment of 5% CO_2_ at 37 °C and utilized between passages 29 and 34. The cultural medium was changed every 48 h until the cells achieved confluency.

#### 2.2.2. Cell Viability and Cytotoxicity

The impact of levan on the viability of HT-29 cells was examined according to the methodology described by [6]. Briefly, the cells were sub-cultured in a 96-well plate, with a density of 20,000 cells per well, and left to adhere for two days. Four different FW2 levan concentrations (0.5, 1, 3, and 5 mg/mL) were prepared. The control consisted of McCoy’s 5A medium, which was supplemented with 10% FBS. The respective treatments (100 μL each) were administered to the corresponding wells and incubated at 37 °C for 24 h. Evaluation of the HT-29 cell viability was conducted using the MTT (methyl thiazolyl tetrazolium; Bio Basic, Markham, ON, Canada) assay. The absorbance was measured at 570 nm, and the results are presented as the mean percentage of cell viability in comparison to the control group (*p* < 0.05).

#### 2.2.3. Antioxidant Activity of FW2 Levan

In order to determine the antioxidative capacity of levan, an ROS assay was conducted on HT-29 cells. These cells were cultured on a 96-well plate (20,000 cells/well) until a confluent monolayer was obtained. To induce ROS production, the cells were initially exposed to IFN-γ (100 ng/mL) for 12 h. Afterwards, the cells were subjected to lipopolysaccharides treatment at a concentration of 100 ng/mL for a duration of 24 h. Subsequently, the cells were co-incubated with levan (2 mg/mL) at 37 °C for 24 h. The control had McCoy’s 5A medium supplemented with 10% FBS. The quantification of ROS expression in response to the treatments was carried out using an intracellular ROS assay kit according to the manufacturer’s instructions (Abcam, Cambridge, UK). For each sample (*n* = 5), the intensity of deep red fluorescence was measured, and the average ROS expression was recorded and represented in a graph.

### 2.3. In Vivo Experiments Using Mice Models

#### 2.3.1. Preparation of Mice Feed

The mice were fed commercially available feed pallets composed of casein (20%), mineral mix (5%), vitamin mix (2%), corn oil (5%), and corn starch (68%) to both the control and experimental groups. However, the experimental group received an additional supplementation of levan EPS. To attain this, purified levan was dissolved in water and incorporated into the feed pallets at a concentration of 3% (*w*/*w*). The blend was thoroughly mixed and subsequently dried to reconstitute the feed pellets and given to the experimental group. In contrast, the control group was provided with the commercial feed pallets in their original form without the addition of any other ingredients.

#### 2.3.2. Animal Models and Dietary Strategy

A total of 12 laboratory mice models (BALB/c), that were five weeks old, were obtained from the animal house facility at the National Institute for Biotechnology and Genetic Engineering (NIBGE), Pakistan. The mice were divided into two groups of six, one of which acted as a control, while the other was the levan treatment group. For initial acclimatization, the animals were provided with the standard diet for a period of five days before the start of the experiment. Following the acclimatization period, the experimental group was provided with the basic diet, which was additionally supplemented with the FW2 levan (3% *w*/*w*), while the control group was fed on an unmodified basic diet. The mice were individually housed in their corresponding cages in a temperature-controlled environment (27 ± 1 °C). Both groups were granted unrestricted availability to feed and water for a duration of 21 days.

#### 2.3.3. Body Weight Analysis

The body weight of mice, in both the control and experimental groups, was measured at two time points: at day zero, i.e., at the start of the experiment, and at day 21, i.e., at the end of the experiment. Mice were carefully positioned on a weighing scale, and their weight changes were noted down in grams.

#### 2.3.4. Determination of Blood Glucose Analysis

The glucose level of each mouse was analyzed using an ACCU-CHEK glucometer (Roche Laboratories, Mannheim Germany) on day 0 and day 21 of the experiment. The values obtained were recorded in mg/dL.

#### 2.3.5. Investigation of Serum Cholesterol Levels

At the end of the 21-day experiment, each mouse was euthanized using Avertin (2,2,2-Tribromoethanol, Sigma-Aldrich, Incheon, Republic of Korea) following the guidelines for handling laboratory animals [27]. Subsequently, blood samples were collected from the mice using Gel & Clot activator tubes (Xinle, Shijiazhuang, Hebei, China). All the tubes were centrifuged instantly at 4 °C and 4000× *g* for 15 min. The resulting serum was carefully separated, collected, and stored at −80 °C for further use. Serum cholesterol levels were analyzed using a Micro Lab-300 chemistry analyzer (Merck, Rahway, NJ, USA) following the manufacturer’s instructions. The recorded cholesterol values were expressed in mg/dL.

### 2.4. Statistical Analyses

The data were presented as mean ± standard deviation (SD). To facilitate inter-sample comparison, a one-way analysis of variance (ANOVA), followed by Tukey’s post hoc analysis, was conducted. Statistical differences were considered significant at *p* < 0.05. A *t*-test was performed when ANOVA was deemed inappropriate.

## 3. Results

### 3.1. Intestinal Cell Viability and Antioxidant Potential in Response to FW2 Levan Exposure

**Figure 1:**
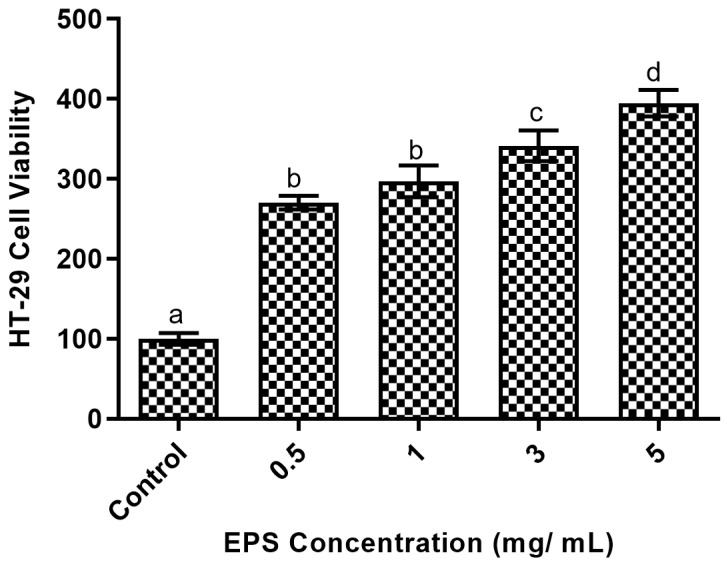
The impact of levan on intestinal epithelial cells’ (HT-29) viability assessed at different concentrations. The data sets (*n* = 5), represented by distinct alphabets, exhibit statistically significant differences (*p* < 0.05).

Levan from *L. reuteri* (FW2) did not exert any cytotoxic effects on the HT-29 cells; instead, it was found to enhance the viability of these cells. All the doses of levan demonstrated a significant increase in cell viability in a concentration-dependent manner, although no significant differences between the 0.5 and 1 mg/mL concentrations were observed. The maximum level of cell viability was observed at 5 mg/ mL levan concentration (Figure 1).

**Figure 2:**
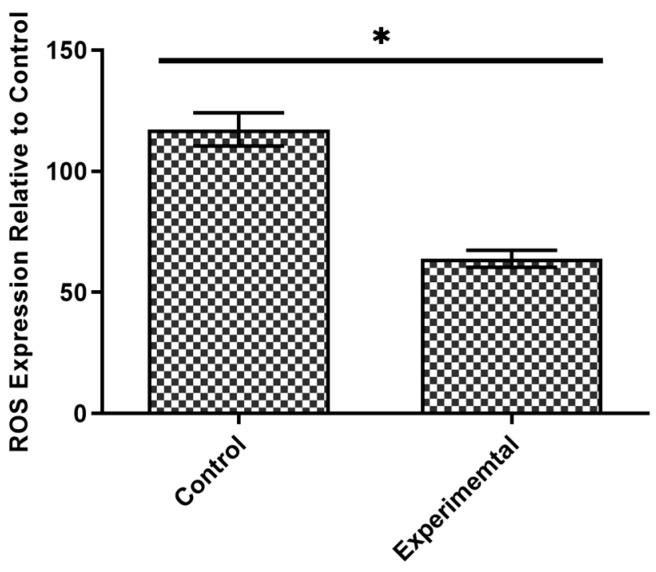
The ROS expression in HT-29 cells treated with levan in comparison with control. (*) indicates a statistically significant difference between the two compared groups (*p* < 0.05, determined by a *t*-test).

Valuably, treatment with levan (2 mg/mL) significantly decreased the oxidative stress in compromised HT-29 cells. Our findings revealed a significant reduction (*p* < 0.05) in the expression of ROS in the damaged HT-29 cells treated with levan compared to the untreated cells, as shown in Figure 2.

### 3.2. Determination of Levan’s Effect on Body Weight

**Figure 3:**
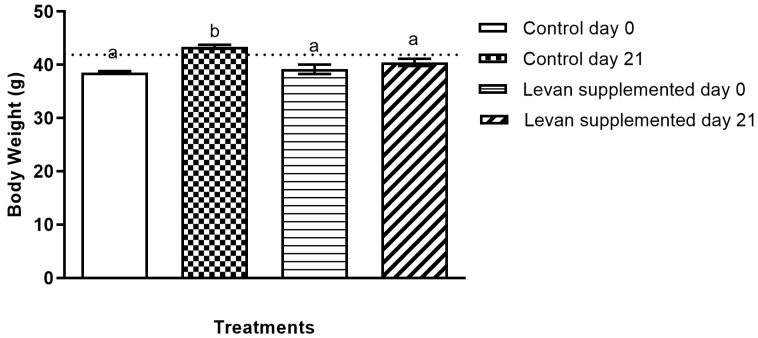
Average body weight gain in the experimental group (basic diet with supplementation of levan) and control group (basic diet) comparing days 0 and 21. The data sets (*n* = 6), represented by distinct alphabets, exhibit statistically significant differences (*p* < 0.05).

To assess the impact of levan on metabolic markers, each mouse of the experimental group was subjected to three weeks of dietary supplementation with levan, and their results were compared with the untreated control group. After the experimental period (day 21), the control group exhibited an average body weight increase of 3.6 g, whereas the levan-fed group showed a weight gain of 2.0 g (Figure 3). The percentage of weight gain was 9.5% for the non-treated mice and 4.7% for the mice treated with levan, indicating that levan is a suitable ingredient for weight management.

### 3.3. Analysis of Blood Glucose Levels in Mice Treated with Levan

**Figure 4:**
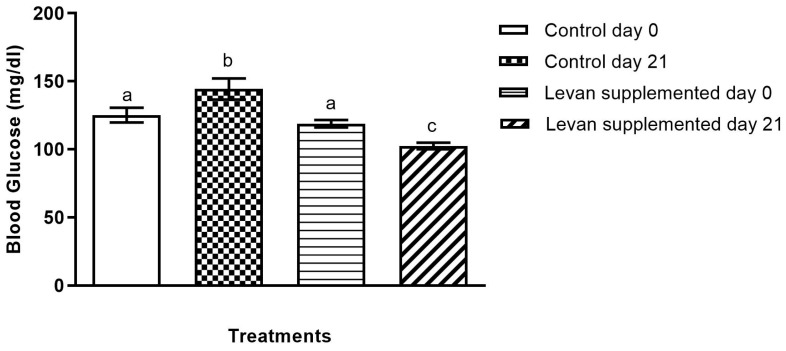
Blood glucose level of levan-supplemented and control group at days 0 and 21. The data sets (*n* = 6), represented by distinct alphabets, exhibit statistically significant differences (*p* < 0.05).

The experiment’s findings revealed a notable disparity in blood glucose levels between the control group and the levan-fed group. Specifically, the blood glucose levels in the control group were increased by 15.2% compared to the initial measurement at the beginning of the experiment. On the other hand, the levan-fed group showed a notable decrease in blood glucose levels, with a reduction of 13.5% compared to the initial measurement (Figure 4). These findings indicate that levan has the potential to regulate blood glucose levels effectively.

### 3.4. Investigation of Serum Cholesterol Levels in Mice Treated with Levan

**Figure 5:**
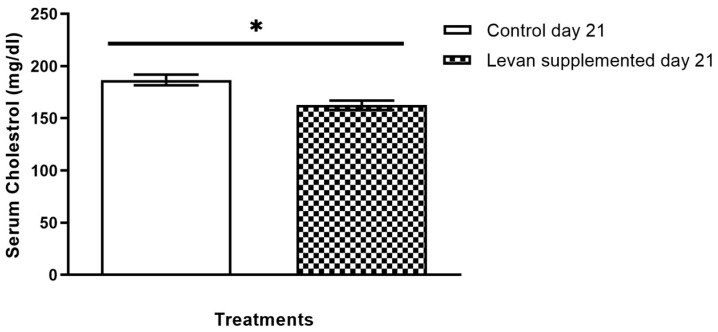
Serum cholesterol level of levan-supplemented and control group in experimental animals. (*) indicates a statistically significant difference between the two compared groups (*p* < 0.05, determined by a *t*-test).

The levan-fed group exhibited lower serum cholesterol levels in comparison with the control group, indicating a prominent hypocholesterolemic effect of levan. In comparison to the control group, levan reduced serum cholesterol levels by 12.63% (Figure 5). These results highlight the potential of levan derived from *L. reuteri* FW2 as a viable serum cholesterol-lowering agent.

## 4. Discussion

In recent years, there has been a significant interest in bacterial EPSs due to their broad range of applications in various industries, including pharmacy, cosmetics, and food. Among these applications, dextran (glucan) extracted from bacteria is the only one that is extensively studied and utilized in the food and pharmaceutical sectors [6,28,29,30]. Until now, there has been limited research available on the biological role of fructans, particularly levans. Our study presents the first evidence of levan from a specific lactic acid bacterium (LAB), *L. reuteri* FW2, exhibiting remarkable viability and antioxidant activity in intestinal epithelial cells.

Modulation of the intestinal epithelial cells by EPSs is one of the important phenomena used to elucidate the significance of their prebiotic mechanisms. An EPS must demonstrate sufficient stability against digestive enzymes in the upper gastrointestinal tract (GIT), as the digested EPSs will not be available for modulating gut microbiota and intestinal epithelial cells. Levans with β-(2→6)-linked fructosyl units are among the non-digestible EPSs which are resistant to hydrolysis by human enzymes [18,31]. Our results showed that levan promotes cell growth of intestinal epithelial cells, without any cytotoxic effect, suggesting its biocompatible nature and potentially safe medicinal agent for intestinal damage. Our findings are in accordance with a recent study that performed an in vitro MTT assay on a normal L-132 cell line to evaluate cell viability and cytotoxic effects by exposing the cells to levan obtained from *Bacillus subtilis* PR-C18. Their findings indicate that PR-C18 levan maintained high cell viability without exerting any cytotoxic effects on L-132 cells [31]. Previously, a study has shown the potential benefit of EPS to modulate the gastrointestinal tract (GIT) by protecting it from toxins [32]. Our findings are further supported by a study showing that EPS116 from *Lactobacillus plantarum* protected the colon and intestines from inflammation and also promoted epithelium cell integrity [33]. The results presented here demonstrate the therapeutic potential of levan in mucosal repair by highlighting its role in promoting epithelial cell proliferation, which is crucial for wound healing after intestinal injury.

EPSs have long been recognized as antioxidant compounds, but it is important to note that their antioxidant properties can be influenced by specific structural features, including monosaccharide content, molecular weight, configuration, and glycosidic bonds [34,35]. Our research findings provide support for the antioxidant capacity of the levan molecule, aligning with previous in vitro and in vivo studies on different microbial EPSs [36,37,38]. The reasons for levan to scavenge free radicals can be associated with the presence of distinct functional groups such as –OH and –CHO, and the nature of glycosidic bonds that donate electrons [31], resulting in the alleviation of reactive oxygen species formed by damaged HT-29 cells in oxidative stress. Additionally, previous reports have shown that fructan supplementation offers protection against oxidative stress and activates the detoxification system [39]. Thus, levan has the potential to serve as a potent natural antioxidant. A previous study has demonstrated that probiotic *L. reuteri* JN101 displayed enhanced adhesion capacity to HT-29 cells in the presence of levan, highlighting the combined multifaceted benefits of levan and its producing probiotic strain on colon epithelial cells [40].

EPSs have also demonstrated effectiveness in regulating symptoms related to obesity and diabetes. A recent study found that a glucan-type EPS reduced serum cholesterol levels and improved the blood glucose profiles in mice [6]. In terms of fructans, extensive research has been conducted on the effects of inulin and fructooligosaccharides on glucose and lipid metabolism [41,42,43,44]. However, the effects of levan in in vivo experiments have not been studied to the same extent as those of fructooligosaccharides and inulin. A recent study by Jang and Kim [45] showed that a combination of poly-γ-glutamic acid and levan improved obesity and dyslipidemia in mice fed a high-fat diet. Additionally, in a separate study, the consumption of levan by Korean women over 12 weeks resulted in a significant decrease in body weight [46]. The plausible reason for this is levan’s selective fermentation by the gut microbiota and production of SCFAs, which alleviate the symptoms of metabolic syndrome. This is supported by a previous study in which mice were fed a high-fat diet supplemented with sodium acetate, propionate, and butyrate SCFAs for 35 days. Their results demonstrated that SCFAs reduced visceral fat mass and weight gain and regulated metabolic processes such as glucose, plasma cholesterol, and free fatty acids [47]. Similarly, our findings indicate that the supplementation of *L. reuteri*-derived levan may help prevent diet-induced weight gain.

The decrease in the glucose level in the *L. reuteri* levan-fed group suggests that levan may possess hypoglycemic properties, making it a promising candidate for managing blood glucose levels. A decrease in blood glucose and serum cholesterol levels was previously reported in rats by using levan supplementation [48,49,50]. In another study, levan-supplemented food was found to reduce serum triglycerides and cholesterol in rats [51]. Overall, our findings demonstrate that levan supplementation in diets may support metabolic health, particularly in managing conditions like high cholesterol, obesity, and diabetes for individuals at risk of metabolic disorders. However, further research and studies are warranted to explore the underlying mechanisms and potential therapeutic applications of levan as a hypoglycemic agent.

## 5. Conclusions

This study investigates the health-beneficial markers of a levan-type EPS derived from a fish gut isolate *L. reuteri* FW2. Based on our results, feed supplementation with levan regulated body weight, blood glucose levels, and serum cholesterol in experimental mice while also improving cell viability and reducing ROS levels in intestinal epithelial cells. For a better comprehension of its mechanism of action demonstrating its prebiotic potential, additional research is necessary to investigate the impact of microbial levan on the modulation of gut microbiota and its relationship to metabolic syndrome. Studies, particularly involving clinical trials or the demography of hyperglycemic and overweight patients, are necessary to explore the health benefits of levan further.

## Data Availability

The original contributions presented in this study are included in the article; further inquiries can be directed to the corresponding authors.

## References

[1] Eckburg P.B., Bik E.M., Bernstein C.N., Purdom E., Dethlefsen L., Sargent M., Relman D.A. (2005). Diversity of the human intestinal microbial flora. Science.

[2] Xiong W. (2022). Intestinal microbiota in various animals. Integr. Zool..

[3] Flint H.J., Duncan S.H., Scott K.P., Louis P. (2007). Interactions and competition within the microbial community of the human colon: Links between diet and health. Environ. Microb..

[4] Meyer D. (2015). Chapter Two—Health benefits of prebiotic fibers. Adv. Food Nutr. Res..

[5] Roberfroid M. (2007). Prebiotics: The concept revisited. J. Nutr..

[6] Ahmad W., Boyajian J.L., Abosalha A., Nasir A., Ashfaq I., Islam P., Schaly S., Thareja R., Hayat A., Rehman M.u. (2022). High-Molecular-Weight Dextran-Type Exopolysaccharide Produced by the Novel *Apilactobacillus waqarii* Improves Metabolic Syndrome: In Vitro and In Vivo Analyses. Int. J. Mol. Sci..

[7] Chou W.T., Sheih I.C., Fang T.J. (2013). The applications of polysaccharides from various mushroom wastes as prebiotics in different systems. J. Food Sci..

[8] Jawad I., Bin Tawseen H., Irfan M., Ahmad W., Hassan M., Sattar F., Akhtar K., Anwar M.A. (2023). Dietary Supplementation of Microbial Dextran and Inulin Exerts Hypocholesterolemic Effects and Modulates Gut Microbiota in BALB/c Mice Models. Int. J. Mol. Sci..

[9] Teferra T.F. (2021). Possible actions of inulin as prebiotic polysaccharide: A review. Food Front..

[10] Domżał-Kędzia M., Ostrowska M., Lewińska A., Łukaszewicz M. (2023). Recent Developments and Applications of Microbial Levan, A Versatile Polysaccharide-Based Biopolymer. Molecules.

[11] Ashfaq I., Amjad H., Ahmad W., Nasir A., Ali A., Ali W.R., Khaliq S., Hayat A., Ali H., Sattar F. (2020). Growth Inhibition of Common Enteric Pathogens in the Intestine of Broilers by Microbially Produced Dextran and Levan Exopolysaccharides. Curr. Microbiol..

[12] Öner E.T., Hernández L., Combie J. (2016). Review of levan polysaccharide: From a century of past experiences to future prospects. Biotechnol. Adv..

[13] Nasir A., Sattar F., Ashfaq I., Lindemann S.R., Chen M.-H., Van den Ende W., Toksoy E., Kirtel O., Khaliq S., Ghauri M.A. (2020). Production and characterization of a high molecular weight levan and fructooligosaccharides from a rhizospheric isolate of *Bacillus aryabhattai*. LWT.

[14] Vijn I., Smeekens S. (1999). Fructan: More than a reserve carbohydrate?. Plant Physiol..

[15] Srikanth R., Reddy C.H.S., Siddartha G., Ramaiah M.J., Uppuluri K.B. (2015). Review on production, characterization and applications of microbial levan. Carbohydr. Polym..

[16] Hamdy A.A., Elattal N.A., Amin M.A., Ali A.E., Mansour N.M., Awad G.E. (2017). Possible correlation between levansucrase production and probiotic activity *of Bacillus sp*. isolated from honey and honey bee. World J. Microbiol. Biotechnol..

[17] Klaewkla M., Pichyangkura R., Charoenwongpaiboon T., Wangpaiboon K., Chunsrivirot S. (2020). Computational design of oligosaccharide producing levansucrase from *Bacillus licheniformis* RN-01 to improve its thermostability for production of levan-type fructooligosaccharides from sucrose. Int. J. Biol. Macromol..

[18] Xu M., Pan L., Wang B., Zou X., Zhang A., Zhou Z., Han Y. (2023). Simulated Digestion and Fecal Fermentation Behaviors of Levan and Its Impacts on the Gut Microbiota. J. Agric. Food Chem..

[19] Kovanda L., Zhang W., Wei X., Luo J., Wu X., Atwill E.R., Vaessen S., Li X., Liu Y. (2019). In Vitro Antimicrobial Activities of Organic Acids and Their Derivatives on Several Species of Gram-Negative and Gram-Positive Bacteria. Molecules.

[20] Fusco W., Lorenzo M.B., Cintoni M., Porcari S., Rinninella E., Kaitsas F., Lener E., Mele M.C., Gasbarrini A., Collado M.C. (2023). Short-Chain Fatty-Acid-Producing Bacteria: Key Components of the Human Gut Microbiota. Nutrients.

[21] Dahech I., Belghith K.S., Hamden K., Feki A., Belghith H., Mejdoub H. (2011). Antidiabetic activity of levan polysaccharide in alloxan-induced diabetic rats. Int. J. Biol. Macromol..

[22] Dahech I., Belghith K.S., Hamden K., Feki A., Belghith H., Mejdoub H. (2011). Oral administration of levan polysaccharide reduces the alloxan-induced oxidative stress in rats. Int. J. Biol. Macromol..

[23] Dal Bello F., Walter J., Hertel C., Hammes W.P.J.S. (2001). *In vitro* study of prebiotic properties of levan-type exopolysaccharides from lactobacilli and non-digestible carbohydrates using denaturing gradient gel electrophoresis. Syst. Appl. Microbiol..

[24] Marx S.P., Winkler S., Hartmeier W. (2000). Metabolization of β-(2, 6)-linked fructose-oligosaccharides by different bifidobacteria. FEMS Microbiol. Lett..

[25] Ahmad W., Nasir A., Sattar F., Ashfaq I., Chen M.-H., Hayat A., Rehman M.U., Zhao S., Khaliq S., Ghauri M.A. (2022). Production of bimodal molecular weight levan by a *Lactobacillus reuteri* isolate from fish gut. Folia Microbiol..

[26] Nasir A., Ahmad W., Sattar F., Ashfaq I., Lindemann S.R., Chen M.-H., Van den Ende W., Toksoy E., Kirtel O., Khaliq S. (2022). Production of a high molecular weight levan by *Bacillus paralicheniformis*, an industrially and agriculturally important isolate from the buffalo grass rhizosphere. Anton. van Leeuwen..

[27] Voipio H.-M., Baneux P., De Segura I.A.G., Hau J., Wolfensohn S. (2008). Guidelines for the veterinary care of laboratory animals: Report of the FELASA/ECLAM/ESLAV Joint Working Group on Veterinary Care. Lab. Anim..

[28] Bhavani A.L., Nisha J. (2010). Dextran—The polysaccharide with versatile uses. Int. J. Pharm. Bio Sci..

[29] Du R., Qiao X., Zhao F., Song Q., Zhou Q., Wang Y., Zhou Z. (2018). Purification, characterization and antioxidant activity of dextran produced by *Leuconostoc pseudomesenteroides* from homemade wine. Carbohydr. Polym..

[30] Soeiro V.C., Melo K.R., Alves M.G., Medeiros M.J., Grilo M.L., Almeida-Lima J., Rocha H.A. (2016). Dextran: Influence of molecular weight in antioxidant properties and immunomodulatory potential. Int. J. Mol. Sci..

[31] Thomas J., Roy P., Ghosh A., Mete M., Sil S.K., Das D. (2024). Prebiotic levan type fructan from *Bacillus subtilis* PR-C18 as a potent antibiofilm agent: Structural elucidation and *in silico* analysis. Carbohydr. Res..

[32] Saadat Y.R., Khosroushahi A.Y., Gargari B.P. (2019). A comprehensive review of anticancer, immunomodulatory and health beneficial effects of the lactic acid bacteria exopolysaccharides. Carbohydr. Polym..

[33] Zhou X., Qi W., Hong T., Xiong T., Gong D., Xie M., Nie S. (2018). Exopolysaccharides from *Lactobacillus plantarum* NCU116 regulate intestinal barrier function via STAT3 signaling pathway. J. Agric. Food Chem..

[34] Andrew M., Jayaraman G. (2020). Structural features of microbial exopolysaccharides in relation to their antioxidant activity. Carbohydr. Res..

[35] Pei F., Ma Y., Chen X., Liu H. (2020). Purification and structural characterization and antioxidant activity of levan from *Bacillus megaterium* PFY-147. Int. J. Biol. Macromol..

[36] Kavitake D., Veerabhadrappa B., Sudharshan S., Kandasamy S., Devi P.B., Dyavaiah M., Shetty P.H. (2022). Oxidative stress alleviating potential of galactan exopolysaccharide from *Weissella confusa* KR780676 in yeast model system. Sci. Rep..

[37] Şengül N., Işık S., Aslım B., Uçar G., Demirbağ A.E. (2011). The effect of exopolysaccharide-producing probiotic strains on gut oxidative damage in experimental colitis. Digest. Dis. Sci..

[38] Xu X., Peng Q., Zhang Y., Tian D., Zhang P., Huang Y., Shi B. (2020). A novel exopolysaccharide produced *by Lactobacillus coryniformis* NA-3 exhibits antioxidant and biofilm-inhibiting properties in vitro. Food Nutr. Res..

[39] Busserolles J.r.m., Gueux E., Rock E., Demigne C., Mazur A., Rayssiguier Y. (2003). Oligofructose protects against the hypertriglyceridemic and pro-oxidative effects of a high fructose diet in rats. J. Nutr..

[40] Cai G., Wu D., Li X., Lu J. (2020). Levan from *Bacillus amyloliquefaciens* JN4 acts as a prebiotic for enhancing the intestinal adhesion capacity of *Lactobacillus reuteri* JN101. Int. J. Biol. Macromol..

[41] Andersson H.B., Ellegård L.H., Bosaeus I.G. (1999). Nondigestibility characteristics of inulin and oligofructose in humans. J. Nutr..

[42] Chambers E.S., Byrne C.S., Morrison D.J., Murphy K.G., Preston T., Tedford C., Holmes E. (2019). Dietary supplementation with inulin-propionate ester or inulin improves insulin sensitivity in adults with overweight and obesity with distinct effects on the gut microbiota, plasma metabolome and systemic inflammatory responses: A randomised cross-over trial. Gut.

[43] Mistry R.H., Gu F., Schols H.A., Verkade H.J., Tietge U.J. (2018). Effect of the prebiotic fiber inulin on cholesterol metabolism in wildtype mice. Sci. Rep..

[44] Rao M., Gao C., Xu L., Jiang L., Zhu J., Chen G., Xu Y. (2019). Effect of inulin-type carbohydrates on insulin resistance in patients with type 2 diabetes and obesity: A systematic review and meta-analysis. J. Diabetes Res..

[45] Jang K.-H., Kim M.-J. (2022). A mixture of poly-γ-glutamic acid and levan ameliorates obesity in high fat diet-induced mice. Food Sci. Biotechnol..

[46] Kang S.-A., Jang K.-H., Lee J.-C., Chang B.-I., Lim Y.-A., Song B.-C. (2003). The effects of fructose polymer levan on the body fat accumulation and serum lipid profiles of Korean women. Korean J. Community Nutr..

[47] Jiao A., Yu B., He J., Yu J., Zheng P., Luo Y., Luo J., Yan H., Wang Q., Wang H. (2021). Sodium acetate, propionate, and butyrate reduce fat accumulation in mice via modulating appetite and relevant genes. Nutrition.

[48] Bahroudi S., Shabanpour B., Combie J., Shabani A., Salimi M. (2020). Levan exerts health benefit effect through alteration in bifidobacteria population. Iranian Biomed. J..

[49] Belghith K.S., Dahech I., Hamden K., Feki A., Mejdoub H., Belghith H. (2012). Hypolipidemic effect of diet supplementation with bacterial levan in cholesterol-fed rats. Int. J. Biol. Macromol..

[50] Dahech I., Harrabi B., Hamden K., Feki A., Mejdoub H., Belghith H., Belghith K.S. (2013). Antioxidant effect of nondigestible levan and its impact on cardiovascular disease and atherosclerosis. Int. J. Biol. Macromol..

[51] Kang S.-A., Hong K.-H., Jang K.-H., Kim S.-H., Lee K.-H., Chang B.-I., Choue R.-W. (2004). Anti-obesity and hypolipidemic effects of dietary levan in high fat diet-induced obese rats. J. Microbiol. Biotechnol..

[52] Burnett L.C., Lunn G., Coico R. (2009). Biosafety: Guidelines for working with pathogenic and infectious microorganisms. Curr. Protoc. Microbiol..

